# Coupled nanowire-based hybrid plasmonic nanocavities on thin substrates

**DOI:** 10.1186/1556-276X-9-641

**Published:** 2014-11-28

**Authors:** Pi-Ju Cheng, Chih-Kai Chiang, Yi-Cheng Chung, Chung-Hao Tien, Tzy-Rong Lin

**Affiliations:** 1 Research Center for Applied Sciences, Academia Sinica, 11529 Taipei, Taiwan; 2 Department of Photonics, National Chiao Tung University, 30010 Hsinchu, Taiwan; 3 Institute of Optoelectronic Sciences, National Taiwan Ocean University, 20224 Keelung, Taiwan; 4 Department of Mechanical and Mechatronic Engineering, National Taiwan Ocean University, 20224 Keelung, Taiwan

**Keywords:** Laser reonators, Surface plasmons, Semiconductor lasers, Waveguides, Nanowires, Nanomembrane

## Abstract

We theoretically analyze nanowire-based hybrid plasmonic nanocavities on thin substrates at visible wavelengths. In the presence of thin suspended substrates, the hybrid plasmonic modes, formed by the coupling between a metal nanowire and a dielectric nanowire with optical gain, exhibit negligible substrate-mediated characteristics and overlap better with the gain region. Consequently, the confinement factor of the guided hybrid modes is enhanced by more than 42%. However, the presence of significant mirror loss remains the main challenge to lasing. By adding silver coatings with a sufficient thickness range on the two end facets, we show that the reflectivity is substantially enhanced to above 50%. For a coating thickness of 50 nm and cavity length of about 4 *μ*m, the quality factor is above 100.

## Background

Nanophotonic technologies based on nanowires have attracted much attention in the last decade. Owing to their interesting optical and electronic properties, nanowires (NWs) could serve as building blocks for novel miniaturized photonic and optoelectronic devices [1] with applications ranging from waveguiding [2-6] to lasing [7-11]. Recently, semiconductor NW-based nanolasers that utilize the surface plasmon polariton (SPP) effect as the optical guiding mechanism have been successfully demonstrated [8,10]. As hybrid plasmonic structures play an important role in determining the modal behavior and lasing properties, the unavoidable metal-based nanostructure significantly increases the fabrication complexity. Cutting-edge bottom-up synthesis techniques [12,13] enable the fabrication of various kinds of NWs with accurately controlled components, dimensions, and shapes. In this study, we analyze the guiding properties of an aligned NW pair formed by a gain NW and a metal NW, fabricated using bottom-up techniques, on a suspended dielectric substrate. We then propose a three-dimensional (3D) plasmonic Fabry-Pérot (FP) nanocavity composed of the NW pair (composed of silver and gallium nitride) truncated by two Ag-coated end facets that act as reflectors. The proposed nanocavity, which is based on the SPP modes at visible wavelengths around 450 nm, is suspended on a thin substrate of thickness *t*. The air gap distance *d* (between the two NWs) and thickness *t* are varied in later calculations under different metal NW radii *r*_m_ and dielectric NW radii *r*_GaN_. We are particularly interested in the case of a thin substrate (*t*=5 nm) with low refractive index (*n*_s_=1.5) corresponding to a free-standing dielectric nanomembrane consisting of, for example, silicon dioxide (SiO _2_). In this way, the bottom-up approaches for organic/inorganic nanomembranes [14,15] may be further integrated with surface plasmonics to enable more functionalities. In addition, the substrate thickness affects the characteristics of the lasing modes. The SPP modes formed from strong coupling between the metal and dielectric NWs on thick substrates often feature substrate-mediated characteristics [16], which result in strong field enhancement at the interface between the substrate and metal region. The significant loss at the metal region and the low overlap with the gain region are therefore the main challenges for lasing. In contrast, hybrid plasmonic modes on a thin substrate often exhibit characteristics of dielectric NW-guided modes and lead to better confinement and lower modal loss. Except for the analysis of modal characteristics by two-dimensional (2D) finite-element method (FEM) [17,18], we utilize 3D FEM to solve for the modal volume *V*_m_ and reflection field pattern. The orthogonality theorem of waveguide modes is applied to extract the modal reflectivity *R*[19]. We also estimate the required cavity length *L*, quality factor *Q*_FP_, and threshold gain *g*_th_ necessary for the lasing action at the target wavelength of 450 nm.

## Methods

**Figure 1:**
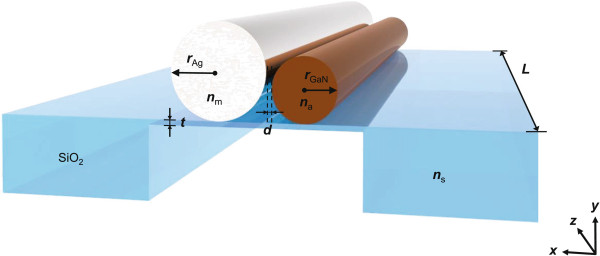
**Schematic of proposed nanocavity.** Aligned nanowire pair on a thin free-standing SiO _2_ substrate. One nanowire consists of Ag, and the other consists of GaN.

In this study, we analyzed an FP nanocavity atop a thin free-standing substrate. We employed FEM to solve Maxwell’s equations for a complex optical system of the proposed structure shown in Figure 1. As a starting point, we adopted the 2D FEM eigensolver of the COMSOL software [20] to investigate the guiding behavior of a waveguide of the proposed configuration without truncation at the two ends. We varied the Ag NW radii *r*_Ag_ and air gap width *d* and studied the corresponding variations in the modal characteristics of the hybrid modes for substrate thicknesses *t*=5 or 500 nm. The modal loss *α*_i_ corresponding to the attenuation of propagating modes was obtained from the imaginary parts of the effective refractive indices under different conditions.We calculated the waveguide confinement factor *Γ*_wg_ as well as the mode area *A*_m_ from the transverse spatial distribution of mode profiles [21]. The transparency threshold *g*_tr_, defined as the ratio of *α*_i_ to *Γ*_wg_, is the minimum material gain necessary to sustain the propagation of the mode without attenuation. In the calculation, we set the refractive index of the metal NW *n*_m_ as that of Ag (0.04+2.65*i*) and the *n*_a_ of GaN as 2.50 at the target wavelength of 450 nm. We utilized SiO _2_ as the low-index substrate material with the refractive index *n*_s_=1.5.

In the following analysis of the plasmonic FP cavity, the resonance mode of an FP cavity is approximated as the standing wave corresponding to the specific transverse-guided mode. We performed 3D FEM to calculate the overall fields (both incident and reflected) inside the cavity. The modal reflection coefficients, relative phase shifts, and reflectivities *R* at the Ag reflectors are extracted from the orthogonality theorem [19]. In order to sustain sufficient modal gain for lasing, we coated the waveguide with Ag layers of thickness 50 nm at the end facets. When compared to the configuration of bare waveguide/air interface, the Ag mirrors significantly increase the reflectivity and decrease the mirror loss. In addition to the reflectivity, the cavity length *L* is another relevant parameter for lasing. We deduced each cavity length that satisfies the FP round-trip phase-matching condition at the target wavelength. By collecting the information about material loss and mirror loss, we can reasonably estimate the quality factor *Q*_FP_ and threshold gain *g*_th_ from the FP formulae [17-19].

In addition, we evaluated the quality factor *Q* of the configurations on substrates of two different thicknesses at a specific value of *L* in a more intuitive but computationally consuming way to verify *Q*_FP_. In 3D FEM calculations, we excited the nanocavity with a ŷ-polarized plane wave. The spatial integration of the squared magnitude of the electric field (proportional to electric energy density) inside the gain region was then recorded when the wavelength was altered through resonance. The *Q* factor was solved from the ratio between the full width at half maximum (FWHM) and the peak resonance wavelengths of the corresponding lineshape. Finally, resolving the field distributions of the resonance modes enables us to determine the mode volume *V*_m_[22].

## Results and discussion

### Modal analysis

The hybrid plasmonic modes of the structure in Figure 1 are hybridized from the surface plasmonic guided modes of the metallic NW and the guided modes of the dielectric NW. According to [16], when a metallic cylinder is in the proximity of a plane, the guided modes TM _0_ and HE _±1_ are coupled and significantly mediated by the polarized charges on the surface of the planar structure. In the entire guiding structure, if a considerable amount of the field is distributed between the metallic region and substrate, the field may not sufficiently overlap with the dielectric NW. We hence explored the modal characteristics of the aligned NW pair with a sufficient air gap width *d* and two distinct substrate thicknesses *t*=5 and 500 nm. The coupling strengths of the fundamental hybrid modes between the two categories of modes are sensitive to variations in the NW radii, *r*_GaN_ and *r*_Ag_, and the width of the air gap *d*. Depending on the parameters, the features of each type of mode can be quite different.

**Figure 2:**
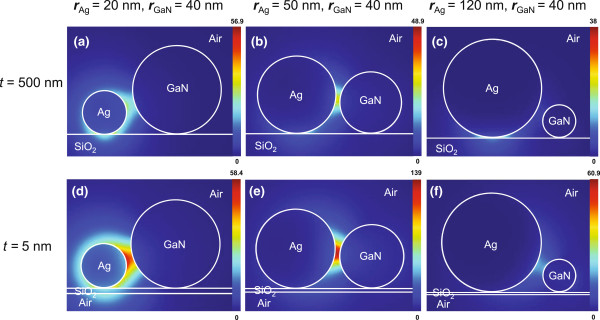
**Cross-sectional modal profiles.** Field magnitudes |**E**(**ρ**)| for the fundamental hybrid plasmonic mode on thick substrates (*t*=500 nm), at a fixed *r*_GaN_= 40 nm and different *r*_Ag_=**(a)** 20 nm, **(b)** 50 nm and **(c)** 120 nm. **(d)**, **(e)**, and **(f)** are the counterparts of **(a)**, **(b)**, and **(c)** for thin substrates (*t*=5 nm).

Figure 2 shows the magnitudes |**E**(**ρ**)| of the cross-sectional profiles of the fundamental hybrid plasmonic modes. At a fixed *r*_GaN_=40 nm, we set *r*_Ag_ to be 20, 50, and 120 nm for substrate thicknesses *t*=5 or 500 nm. In Figure 2a,d, at the smaller *r*_Ag_ (<*r*_GaN_), hybrid modes on both substrates show a significant distribution over the metallic (lossy) region. At larger *r*_Ag_ (>*r*_GaN_) as shown in Figure 2f, the cross-sectional field profiles of hybrid modes on the thin substrate (*t*=5 nm) otherwise extensively overlap with the gain region, yet the modes on the thick substrate (*t*=500 nm) strongly localize at the bottom of the metallic NW [23] as shown in Figure 2c. When *r*_GaN_≈*r*_Ag_ as shown in Figure 2b,e, the fields are mainly distributed within the air gap regions. Although the hybrid modes in Figure 2b,e have similar field distributions, the components of the hybrid modes originating from the guiding modes of individual NW have different weightings because of the mediation of the substrates. Even though the closest distance between the circular surfaces of the two NW pairs increases as the difference in radii increases, the effect of distance occurs only while *r*_Ag_>*r*_GaN_.

**Figure 3:**
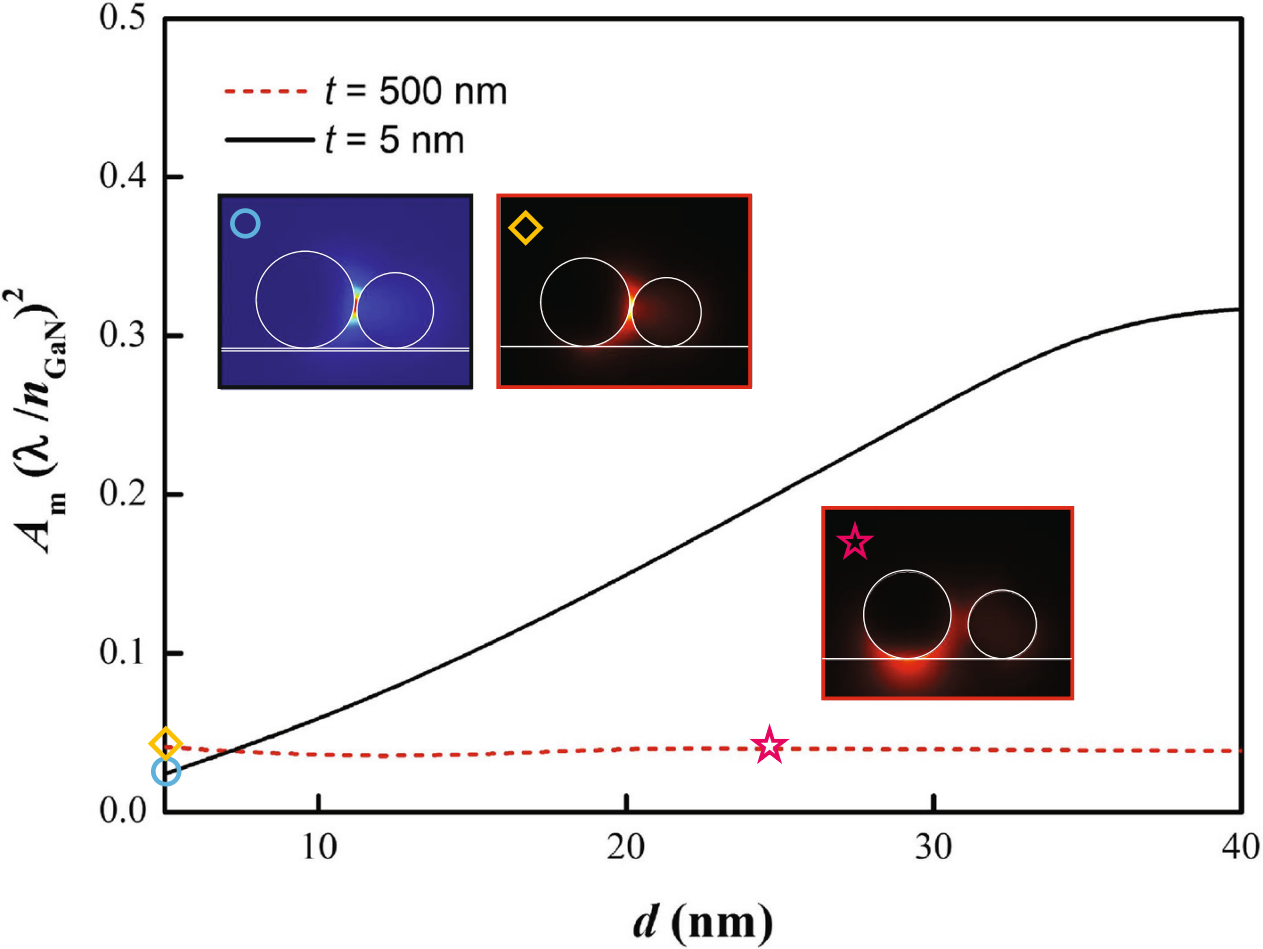
**Mode areas on thin and thick substrates at various gap widths*d*.** The insets are mode profiles corresponding to different *d* and substrate thicknesses *t*. The insets marked by star, circle, and rhombus signs correspond, respectively, to *t*=500 nm and *d*=25 nm; *t*=5 nm and *d*=5 nm; and *t*=500 nm and *d*=5 nm.

We evaluated the mode areas of various air gap widths for both substrate thicknesses, as shown in Figure 3. The fundamental hybrid mode areas on thin substrates, formed by the coupling of the HE _11_ modes of the dielectric NWs and the TM _0_ modes of metallic NWs, are proportional to the air gap widths. On the other hand, the mode areas on thick substrates are not affected by the distance *d* between the two NWs. The maxima of mode profiles are located below the metallic NWs. In other words, the hybrid modes on the thick substrate are manipulated by the substrate-mediated plasmonic modes around the metallic NW and suffer from higher modal loss, as will be shown in the following discussion.

**Figure 4:**
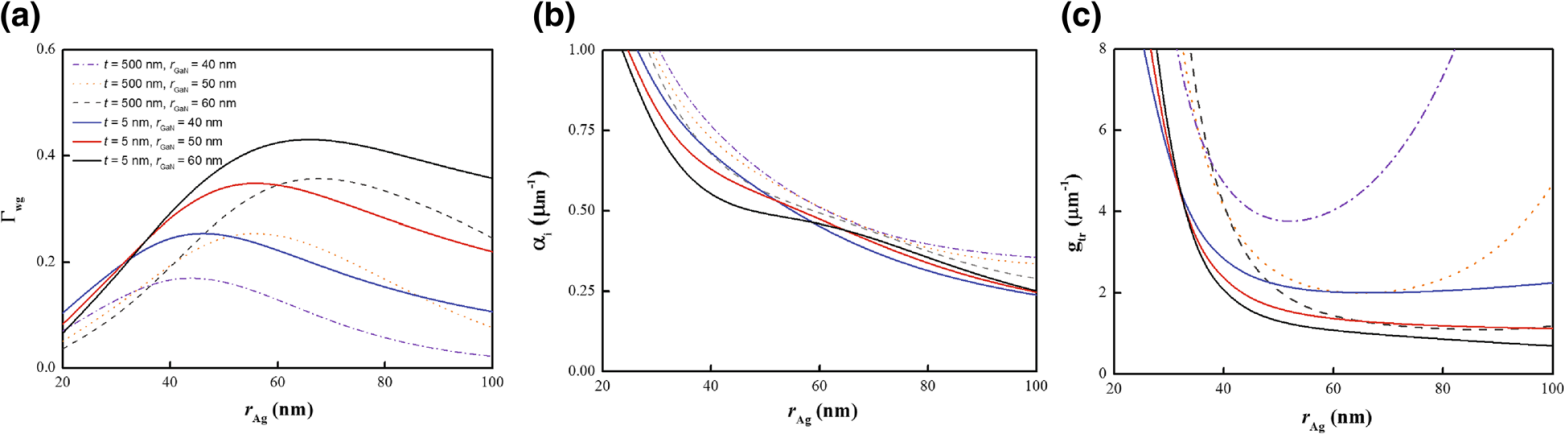
**The waveguide confinement factor, modal loss, and transparency threshold gain versus*r***_**Ag**_**.(a)** The waveguide confinement factor *Γ*_wg_, **(b)** modal loss *α*_i_, and **(c)** transparency threshold gain *g*_tr_ of the fundamental hybrid plasmonic modes as a function of *r*_Ag_ for *r*_GaN_= 40, 50, or 60 nm on thick (*t*=500 nm) and thin substrate (*t*=5 nm).

The evolution of different modes as *r*_Ag_ varies is more easily understood from the waveguide confinement factor *Γ*_wg_ and modal loss *α*_i_, which are relevant to lasing. In Figure 4a,b,c, we numerically solved for *Γ*_wg_, *α*_i_, and the corresponding transparency threshold gain *g*_tr_ of the fundamental hybrid mode as a function of the metallic wire radii *r*_Ag_ at different dielectric wire radii *r*_GaN_=40, 50, and 60 nm and substrate thicknesses *t*=5 and 500 nm. From Figures 4a,b, although *r*_GaN_ has a significant impact on *Γ*_wg_, it does not significantly change *α*_i_. When *r*_Ag_ is small, the hybrid mode is distributed over the entire metallic NW, which further increases *α*_i_ as a result. On the other hand, when *r*_Ag_ is large, the drop in the *Γ*_wg_ mainly results from the increase in the closest distance between the two NWs. For a relatively large *r*_Ag_ and thick substrate, the field does not penetrate significantly into the metal region; instead, it is locally enhanced at the bottom of the metal wire as a response to the induced substrate charges. In contrast, on thin substrates, because of the good coupling to the guiding modes of the gain NW, *Γ*_wg_ of hybrid modes increases as *r*_GaN_ increases. At the smaller substrate thicknesses, as shown in Figure 4a,b, *Γ*_wg_ of the hybrid plasmonic mode is increased by over 40% and *α*_i_ is reduced by more than 30% at fixed wire radii (*r*_Ag_=120 nm and *r*_GaN_=60 nm). Figure 4c shows that the *g*_tr_ of the configuration on the thin substrate is much smaller than that on the thick substrate. In addition to modal loss, the large mirror loss due to the mode mismatching at the waveguide/mirror junction is another factor hindering lasing action.

### Cavity design

**Figure 5:**
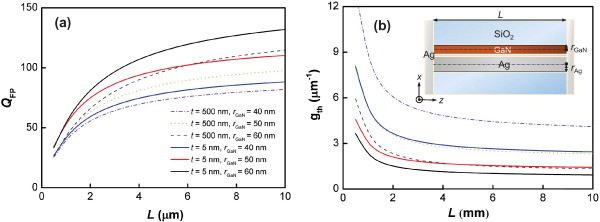
**Quality factor and threshold gain of the fundamental hybrid mode versus cavity length.(a)** Quality factor *Q*_FP_ and **(b)** threshold gain *g*_th_ of the fundamental hybrid mode as a function of cavity length *L* for *r*_GaN_= 40, 50, and 60 nm on thick (*t*=500 nm) and thin substrates (*t*=5 nm). An increase in *Q*_FP_ of 20% for thin substrates is achievable at approximately *L*=4 *μ*m. The inset in **(b)** is the top view of the cavity with two end-facet coatings acting as reflectors.

With a cavity length *L* in the sub-micron range, power leakage from the two end facets can be substantial, and increasing the reflectivity becomes necessary for threshold reduction. For this purpose, we considered 50-nm Ag coatings at the two end facets of the hybrid plasmonic nanocavity, as shown in the inset of Figure 5a. Using the orthogonality theorem, we calculated the reflectivities from the standing wave pattern [17,19]. For the configuration on the thin substrate, the reflectivity (*R*=57.5*%*) is 18% larger than that on the thick substrate (*R*=48.6*%*). The corresponding parameters, such as the quality factors and threshold gains of FP resonances, were solved for at different cavity lengths *L*, as shown in Figure 5a,b. Rather than increasing the reflectivity, an alternative solution to decrease the mirror loss is to elongate the FP cavity. At a cavity length as short as a few micrometers (*L*≈4 *μ*m), the *Q* factor exceeds 100 and *g*_th_ is lower than 1 *μm*^-1^. This threshold gain might be potentially sustainable by the gain medium under intensive optical pumping. By observing the corresponding *Q*_FP_ components originating from material loss and mirror loss, we see that they both benefit from the thin substrate configuration. Consequently, for each cavity length, the reduction of modal absorption and the increase of facet reflectivity are significant on decreasing the substrate thickness.

**Figure 6:**
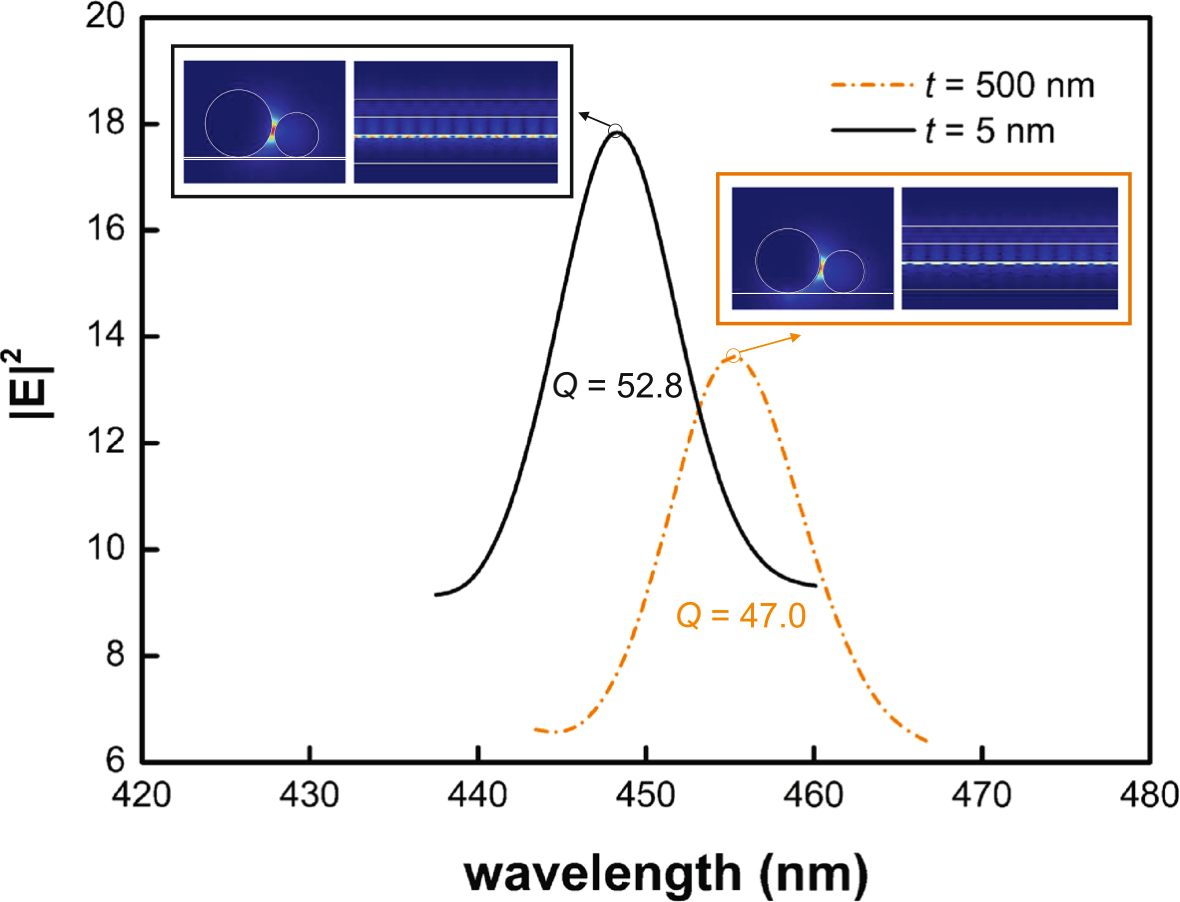
**Resonance lineshapes calculated using 3D FEM.** Resonance lineshapes for the mode (red line) at *L*=1500 nm and *t*= 5 nm and for the mode (black line) at *L*=1500 nm and *t*= 500 nm. The corresponding *Q* factors are approximately 52.75 and 45.00, respectively. The insets are the corresponding field profiles (side views and top views) at the peak resonance wavelengths.

To verify that *Q*_FP_ is a reasonable estimate of the *Q* factor for the hybrid plasmonic mode nanocavity, we also investigated the cavity modes for *L*=1500 nm on a thin substrate with *t*=5 nm and on a thick substrate with *t*=500 nm, as shown in Figure 6. The *Q* factors for cavities on both types of substrates are illustrated in Figure 6. The *Q* factor is approximately 53 for the cavity with *t*=5 nm and approximately 47 for *t*=500 nm, both of which are in good agreement with *Q*_FP_. We then examined the field profiles of the resonance modes at each peak wavelength. The front view (*x*-*y* plane) of the substrate-mediated hybrid plasmonic mode on the thick substrate reveals additional localization below the metallic NW, while that on the thin substrate is well confined inside the air gap and is close to the gain region. We calculated *V*_m_ for both configurations: *V*_m_=0.07 (*λ*_0_/*n*_GaN_)^3^ for *t*=5 nm and *V*_m_=0.1 (*λ*_0_/*n*_GaN_)^3^ for *t*=500 nm. On the thin substrate, the proper cavity design results in a strongly confined mode distribution and hence a smaller mode volume *V*_m_.

## Conclusions

We have proposed and analyzed a novel three-dimensional hybrid plasmonic Fabry-Pérot nanocavity with a metallic and dielectric nanowire pair on a thin substrate. We investigated the effect of the thin substrate on the fundamental hybrid plasmonic modes that exhibit ultrasmall mode areas. By using the finite-element method, we numerically solved for the guided modes of the hybrid plasmonic waveguide at a wavelength of 450 nm. The confinement factors, modal losses, and corresponding transparency thresholds of the guided modes on thin and thick substrates were explored for various wire radii. In comparison with the case of thick substrates (*t*=500 nm), we observed that for thin substrates (*t*=5 nm) with *r*_Ag_=50 nm and *r*_GaN_=40 nm, the modal loss is lower by 10% and the waveguide confinement factor is larger by 50%. While the radii of the NW pair on the thin substrate are comparable, the fundamental hybrid plasmonic mode exhibits superior characteristics to achieve low material loss. To reduce the mirror loss, we additionally considered silver coatings at the two end facets as reflectors.The results show that the reflectivity is substantially enhanced when the substrate thickness is within the nanometer range because of better mode profile matching at the waveguide/reflector interface. Instead of increasing the reflectivity, an alternative solution to decrease the mirror loss is to increase the FP cavity length. At a coating thickness of 50 nm and a cavity length nearly equal to 4 *μ*m, the quality factor is above 100 and the threshold gain is lower than 1 *μm*^-1^. The proposed nanowire-based plasmonic nanocavities on a free-standing nanomembrane are compatible with state-of-the-art bottom-up fabrication technology and could be attractive candidates for active photonic/surface plasmonic systems.

## Competing interests

The authors declare that they have no competing interests.

## Authors’ contributions

PJC, CKC, and TRL developed the concept and the analysis. PJC, CKC, and YCC contributed to the numerical simulation. PJC, CKC, and TRL wrote the manuscript. PJC, CKC, CHT, and TRL discussed the results and commented on the manuscript. All authors read and approved the final manuscript.

## References

[B1] Yang P Yan R Fardy M **Semiconductor nanowire: what’s next?** Nano Lett 2010 10 1529 1536 10.1021/nl100665r 20394412

[B2] Wang Y Ma Y Guo X Tong L **Single-mode plasmonic waveguiding properties of metal nanowires with dielectric substrates** Opt Express 2012 20 19006 19015 10.1364/OE.20.019006 23038541

[B3] Bian Y Zheng Z Zhao X Su Y Lui L Liu J Zhu J Zhou T **Guiding of long-range hybrid plasmon polariton in a coupled nanowire array at deep-subwavelength scale** IEEE Photonics Technol Lett 2012 24 1279 1281

[B4] Ta VD Chen R Sun HD **Wide-range coupling between surface plasmon polariton and cylindrical dielectric waveguide mode** Opt Express 2011 19 13598 13603 10.1364/OE.19.013598 21747515

[B5] Bian Y Zheng Z Zhao X Liu L Liu J Zhu J Zhou T **Nanowire based hybrid plasmonic structures for low-threshold lasing at the subwavelength scale** Opt Commun 2013 287 245 249

[B6] Oulton RF Sorger VJ Genov DA Pile DFP Zhang X **A hybrid plasmonic waveguide for subwavelength confinement and long-range propagation** Nat Photonics 2008 2 496 500 10.1038/nphoton.2008.131

[B7] Duan X Huang Y Agarwal R Lieber CM **Single-nanowire electrically driven lasers** Nature 2003 421 241 245 10.1038/nature01353 12529637

[B8] Oulton RF Sorger VJ Zentgraf T Ma R-M Gladden C Dai L Bartal G Zhang X **Plasmon lasers at deep subwavelength scale** Nature 2009 461 629 632 10.1038/nature08364 19718019

[B9] Russell KJ Hu EL **Gap-mode plasmonic nanocavity** Appl Phys Lett 2010 97 163115 10.1063/1.3505154

[B10] Lu Y-J Kim J Chen H-Y Wu C Dabidian N Sanders CE Wang C-Y Lu M-Y Li B-H Qiu X Chang W-H Chen L-J Shvets G Shih C-K Gwo S **Plasmonic nanolaser using epitaxially grown silver film** Science 2012 337 450 453 10.1126/science.1223504 22837524

[B11] Miyazaki HT Kurokawa Y **Squeezing visible light waves into a 3-nm-thick and 55-nm-long plasmon cavity** Phys Rev Lett 2006 96 097401 16606313 10.1103/PhysRevLett.96.097401

[B12] Fan HM Fan XF Ni ZH Shen ZX Feng YP Zou BS **Orientation-dependent Raman spectroscopy of single wurtzite cds nanowires** J Phys Chem C 2008 112 1865 1870 10.1021/jp7096839

[B13] Bertness KA Sanford NA Davydov AV **Gan nanowires grown by molecular beam epitaxy** IEEE J Sel Top Quantum Electron 2011 17 847 858

[B14] Markutsya S Jiang C Pikus Y Tsukruk VV **Freely suspended layer-by-layer nanomembranes: testing micromechanical properties** Adv Funct Mater 2005 15 771 780 10.1002/adfm.200400149

[B15] Ariga K Hill JP Ji Q **Layer-by-layer assembly as a versatile bottom-up nanofabrication technique for exploratory research and realistic application** Phys Chem Chem Phys 2007 9 2319 2340 10.1039/b700410a 17492095

[B16] Zhang S Xu H **Optimizing substrate-mediated plasmon coupling toward high-performance plasmonic nanowire waveguides** ACS Nano 2012 6 8128 8135 10.1021/nn302755a 22892010

[B17] Chang S-W Lin T-R Chuang SL **Theory of plasmonic Fabry-Perot nanolasers** Opt Express 2010 18 15039 15053 10.1364/OE.18.015039 20639990

[B18] Cheng P-J Weng C-Y Chang S-W Lin T-R Tien C-H **Cladding effect on hybrid plasmonic nanowire cavity at telecommunication wavelengths** IEEE J Sel Top Quantum Electron 2013 19 4800306 4800306

[B19] Cheng P-J Weng C-Y Chang S-W Lin T-R Tien C-H **Plasmonic gap-mode nanocavities with metallic mirrors in high-index cladding** Opt Express 2013 21 13479 13491 10.1364/OE.21.013479 23736601

[B20] **COMSOL Multiphysics** [http://www.comsol.com/]

[B21] Chang S-W Chuang SL **Fundamental formulation for plasmonic nanolasers** IEEE J Quantum Electron 2009 45 1014 1023

[B22] Robinson JT Manolatou C Chen L Lipson M **Ultrasmall mode volumes in dielectric optical microcavities** Phys Rev Lett 2005 95 143901 16241653 10.1103/PhysRevLett.95.143901

[B23] Lin T-R Chang S-W Chuang SL Zhang Z Schuck PJ **Coating effect on optical resonance of plasmonic nanobowtie antenna** Appl Phys Lett 2010 97 063106 10.1063/1.3478228

